# Accelerating Advanced Materials Innovation with New Approach Methodologies

**DOI:** 10.3390/nano16211345

**Published:** 2026-10-23

**Authors:** Susanne Bremer-Hoffmann, Agnieszka Mech, Hubert Rauscher

**Affiliations:** European Commission, Joint Research Centre (JRC), 21027 Ispra, Italy; agnieszka.mech@ec.europa.eu (A.M.); hubert.rauscher@ec.europa.eu (H.R.)

**Keywords:** advanced materials, innovation, safe and sustainable by design, new approach methodologies, in-house qualification, molecular/process redesign

## Abstract

Overcoming the translational gap and accelerating the development of advanced materials require enabling tools that provide reliable information to support decision-making throughout the innovation process. New Approach Methodologies (NAMs) can contribute substantially by generating relevant information while reducing reliance on conventional testing approaches. However, the reliability and relevance of many NAMs have not yet been sufficiently established across the applicability domains relevant to advanced materials. Given their diversity and complexity, formal validation of NAMs across the entire range of materials and applications is unlikely to be realistic. Instead, a fit-for-purpose approach is needed, with the level of evidence and qualification tailored to the intended use and applicability domain of the NAM. During early innovation, NAMs may support exploration, research, and iterative decision-making, where full regulatory qualification may not be necessary. In contrast, NAMs used to support regulatory decisions require more stringent qualification. This perspective paper describes the different roles NAMs can play in innovation and discusses a flexible application of existing qualification criteria according to intended use, supporting efficient and accelerated innovation especially for advanced and complex materials while maintaining confidence in NAM-derived information.

## 1. Introduction

Advanced materials are increasingly recognized as strategic enablers of Europe’s competitiveness, green and digital transition, economic security and technological leadership. Recent European policy initiatives, including the Communication on Advanced Materials for Industrial Leadership [1], the forthcoming Advanced Materials Act [2] and the Competitiveness Compass [3], identify advanced materials as a key technology area requiring accelerated innovation and faster deployment to strengthen industrial resilience and strategic autonomy. Advanced materials underpin technological innovation across strategic sectors, including health, energy, electronics, mobility and construction. At the same time, there is increasing recognition that reducing the time from discovery to market while maintaining high standards of safety and sustainability will require new approaches to generate decision-relevant evidence much earlier in the innovation process compared to the current practice. Achieving this ambition is far from straightforward. Unlike conventional materials, advanced materials are intentionally engineered to achieve novel or enhanced functionalities, often through complex combinations of composition, structure, interfaces, morphology and manufacturing processes. Consequently, material development is rarely a linear process. Instead, innovation proceeds through iterative cycles of design, characterization, testing and redesign, during which multiple decisions must be taken by the innovator before complete evidence on functionality, manufacturability, but also safety and more recent sustainability as well is available. The need to make innovation faster, more cost-effective, and more successful has increased the demand for predictive tools that can reduce the uncertainty during material development, and which allow to overcome the translational gap between early research and market application. A major challenge in innovation is that promising discoveries may fail during development because established models do not reliably predict human responses, leading to delays, high costs, and inefficiencies. Improving the predictivity of innovation pipelines therefore requires the generation of more reliable mechanistic and human-relevant data already early in development. In this context, fit-for-purpose NAMs offer an opportunity to accelerate the innovation process, provided that their suitability is assessed against criteria defined for the specific context of use. Predictive NAMs can support earlier and more informed decision-making by providing human-relevant information and establishing greater confidence in the applicability of alternative methods for safety assessment [4,5,6]. At the same time predictive NAMs can provide a market advantage during the innovation process by decreasing the time-to-market. However, the key question for innovators is whether a NAM generates sufficient confidence to support a particular decision and also whether the decisions made would facilitate regulatory acceptance of the product later [7].

The potential competitive advantage of NAMs in this context is, however, not determined by speed alone. Faster generation of information only translates into a competitive advantage where the resulting capabilities, knowledge or technologies are not equally accessible to all innovators. Intellectual property (IP) rights and the ownership or control of proprietary methods, technologies and associated data may therefore be important determinants of the value of NAMs within innovation. The OECD has highlighted the relevance of intellectual property rights for innovation and the development and diffusion of emerging technologies [8]. It should be said that developers may retain IP rights associated with NAMs they have developed and qualified, whether these are used internally or subsequently commercialised. Where NAM-generated evidence is submitted for regulatory assessment, its disclosure and confidentiality are governed by the applicable sector-specific regulatory requirements. For example, Regulation (EU) 2019/1381 establishes transparency requirements for EU food-chain risk assessment while providing for the protection of certain confidential information, subject to the applicable conditions [9].

The potential of NAMs has also been recognized by the European Commission within the European Commission’s Safe and Sustainable by Design (SSbD) framework. The core concept of the SSbD framework is to ensure safety and sustainability throughout development and along the entire life cycle of chemicals and products and to steer innovation to design or redesign chemicals, materials, processes and products by identifying potential safety issues, sustainability impacts and trade-offs early-on in the innovation process [10]. Rather than assessing risks only once a material or product has reached a mature stage of development, SSbD encourages iterative evidence generation to guide material selection, design optimisation and innovation decisions throughout development [11,12]. In this framework, NAMs can play an important role by addressing safety and sustainability considerations already at the design stage, thereby supporting early decision-making instead of relying on late-stage regulatory assessment [13,14]. Current policies are increasingly addressing the need to integrate innovative tools to accelerate innovation and to help overcome the translational gap. For example, in the proposal for the European Biotech act [15] NAMs are characterised as having the potential to generate scientific and technological data that are comparable to, or in some cases more informative, and generated more rapidly than those obtained through current standard methods. The resulting advantage will contribute to strengthening the innovation ecosystem and enhanced European competitiveness.

Recent EU policy initiatives increasingly recognise the need for innovation-friendly regulatory frameworks across different technological and regulatory domains. On 9 September 2026, the European Commission adopted proposals for the European Innovation and a Council Recommendation establishing common principles for regulatory sandboxes, reflecting a broader, cross-sectoral approach to facilitating innovation and regulatory learning. Regulatory sandbox provisions are also included in existing EU legislation or legislative proposals concerning pharmaceuticals, medical devices and in vitro diagnostics, and biotechnology. By providing controlled environments for experimentation under regulatory supervision, regulatory sandboxes may offer opportunities to explore innovative technologies and methodologies, including NAMs, while maintaining appropriate safeguards. They may also facilitate dialogue between innovators and regulators on the evidence needed to support future regulatory decisions [16,17,18,19,20].

As such, NAMs support the identification of potential hazards, exposure pathways, and environmental impacts during the development of chemicals, materials, and products. Their relevance is not limited to biomedical applications. Nano and Advanced materials developed for sectors such as automotive, aerospace, electronics or energy may be primarily designed to achieve specific technical performance characteristics. However, irrespective of their intended industrial application, their development must also consider potential impacts on human health and the environment, as well as applicable regulatory safety requirements. Focusing exclusively on technical performance and addressing safety only at the final stages of development may reveal concerns when substantial resources have already been invested in material optimisation and scale-up. NAMS enable developers to compare alternatives, optimise material properties, and redesign products before large-scale production or market entry, thereby reducing late-stage failures, costly reformulations, and unforeseen risks. In this way, NAMs can help accelerate innovation while maintaining high levels of protection for human health and the environment.

However, detailed guidance on how NAMs can support decision-making throughout the iterative innovation process remains limited. NAMs may fulfil different functions at different stages of development—from early material screening and optimisation to process development and regulatory safety assessment—and these applications do not necessarily require the same level of qualification or supporting evidence. While a variety of readiness frameworks for assessing the use of NAMs in regulatory contexts already exist [21,22,23], further guidance is needed on which criteria defined in these frameworks are necessary or most appropriate for a given context of use within innovation workflows. Such guidance could help determine the level and type of evidence needed to establish whether a NAM is fit for purpose in a specific application and thereby reduce uncertainty in decision-making.

In this regard, it is increasingly evident that not all criteria defined for the regulatory qualification of NAMs need to be met uniformly across all stages of applications in the innovation pipeline. Rather, the level of confidence, validation and supporting evidence required may depend on the intended use of the NAM, the decision it informs, and the stage of development at which it is applied. Determining which criteria are relevant and what level of supporting evidence is appropriate for different contexts of use therefore remains an important gap.

This perspective aims to initiate a discussion on whether specific guidance is needed for the use of NAMs within innovation workflows. In particular, we discuss whether the criteria established in existing NAM readiness and assessment frameworks need to be applied in their entirety at each stage of innovation, or whether a more context-dependent approach may be appropriate. Such an approach would consider which criteria are most relevant to a specific context of use and decision, and what level of evidence is sufficient to provide confidence in the NAM for that purpose. We propose that addressing these questions could help reduce unnecessary barriers to the earlier and broader use of NAMs in innovation while ensuring that the level of confidence remains proportionate to the decisions being made.

## 2. Results

### 2.1. Application of NAMs in Innovation Processes

During the innovation process, NAMs can support the identification and understanding of potential hazards and thereby inform decision-making on the safe design, handling, use, and disposal of chemicals and materials (Table 1).

Figure 1 illustrates the potential contributions of NAMs throughout the innovation process. At each stage, NAM-derived data can support evidence-based decision-making, enabling the early identification of potential hazards and undesirable characteristics associated with manufacturing processes. The diamond-shaped elements represent decision points at which the available evidence is evaluated to determine the appropriate next step in the innovation process. At each decision point, three questions are considered: (1) What is the context of use—that is, which decision must be informed for which material, endpoint, exposure scenario and stage of development? (2) Is the selected NAM scientifically suitable for this purpose, considering its biological relevance, applicability domain, potential material–assay interference, adequacy of controls, repeatability and associated uncertainty? (3) What are the consequences of the decision, taking into account whether the evidence will inform exploratory screening or a reversible design decision, or a higher-consequence safety, compliance or regulatory decision? Together, these considerations guide whether the available evidence is sufficient to proceed, whether additional information is needed, or whether material or process redesign is warranted. The colored arrows in Figure 1 indicate iterative refinement cycles within the innovation process. If the available information is not sufficient, feedback loops start additional testing. This testing may include the need to develop additional NAMs to identify hazardous mechanisms or previously unknown critical quality attributes (red loop), or to collect more data on safety, functionality, or manufacturing (violet loop). When the available data are sufficient but indicate a hazardous profile or undesirable manufacturing characteristics, molecular and/or process redesign can be initiated (orange and green loops). These iterative refinement cycles continue until sufficient evidence has been generated to support regulatory safety testing and submission to the competent authorities (blue line).

#### 2.1.1. NAMs for Screening and Prioritization

At early stages of innovation, where experimental data are often limited, predictions based on structurally similar substances together with screening-level NAMs can provide important information for initial decision-making. The primary purpose of applying such NAMs during early-stage assessments is not necessarily to make final regulatory decisions, but to identify potential hazards or undesired properties of chemicals and thereby prioritization and further investigation. A well-established example is the U.S. EPA’s ToxCast program, which uses medium- and high-throughput in vitro screening assays to characterize the bioactivity of thousands of chemicals and supports their prioritization for further testing (Box 1). For example, ToxCast data have been used within the EPA’s Endocrine Disruptor Screening Program to prioritize chemicals for further endocrine evaluation and, more recently, to prioritize pesticide registration-review cases where estrogen or androgen receptor bioactivity was identified [24,25]. Although high-throughput screening platforms such as ToxCast can support early hazard prioritisation, their assays and interpretation cannot be directly extrapolated from conventional chemicals to advanced (nano)materials without additional assessment. Unlike many dissolved chemicals, nanomaterials may undergo agglomeration, sedimentation, dissolution, and changes in surface properties in the exposure medium. Adsorption of proteins and other biomolecules can form a biomolecular corona that changes particle identity and interactions with biological systems. Moreover, nanomaterials may interfere with assay components or readouts through light absorption or scattering, fluorescence quenching, adsorption of dyes or reagents, or catalytic activity [26]. These effects can generate apparent biological responses or mask genuine effects, depending on the material and assay. Consequently, early screening should be accompanied, as appropriate to the material and intended decision, by characterisation of the material under relevant exposure conditions, assessment of dispersion stability and dissolution, consideration of the delivered or cellular dose, and material-specific interference controls. Where optical or reagent-based endpoints are susceptible to interference, orthogonal methods based on different measurement principles can help determine whether an observed response reflects biological activity or an assay artefact. These screening tools can be either in silico (computer-based) or in vitro (lab-based). Whereas in silico models use computer simulations to predict a substance’s properties based on its chemical structure, in vitro tests are designed for rapid and cost-effective screening of a vast library of compounds, materials, or formulations to find those that have specific functional, (secondary) pharmacological or toxicological properties [27]. High-throughput in vitro tests rely on assay miniaturization and automated liquid handling to enable rapid screening of advanced materials using small amounts of test material and reagents. This increases testing throughput while reducing costs, material requirements, and experimental variability, making these approaches particularly suitable for the early safety assessment of large libraries of advanced materials [5]. New methodologies that improve the screening and prioritization process offer a significant competitive advantage for the developer who uses them [28,29,30,31]. For example, in drug discovery, a reliable and relevant in vitro method that can quickly and accurately filter millions of ineffective compounds at molecular level saves a vast amount of time and resources during target identification allowing companies to bring new products to market faster. In vitro methods can reduce the risk of pursuing projects that are unlikely to succeed. In research and development, misprioritizing projects can lead to substantial financial losses, so being able to accurately identify the most promising candidates is crucial. A screening tool with a lower false positive or false negative rate provides a competitive edge by ensuring that resources are directed toward the most valuable opportunities.

Box 1 **A Case Study in High-Throughput Screening and NAMs (ToxCast).**

An example of a well-documented and transparent screening programme was ToxCast (Toxicity Forecaster)—a major initiative launched by the U.S. Environmental Protection Agency (EPA) [25]. The program’s goal was not just to say whether a chemical was "toxic," but to identify the specific biological pathways it disrupted [32]. For example, it could pinpoint if a chemical interacted with the estrogen receptor, which could indicate a potential for endocrine disruption. By generating a vast amount of data on thousands of chemicals in a cost-effective way, ToxCast enabled the EPA to prioritize which chemicals needed further, more detailed testing. It provided a scientific basis for identifying chemicals that were most likely to pose a risk and therefore required a closer look. ToxCast utilized a battery of hundreds of in vitro assays. These assays, often automated, were designed to quickly measure how a chemical affects a variety of biological processes and pathways, such as hormonal signalling, enzyme activity, and cell growth. The selection of an assay included in the screening programme was dependent on its ability to meet rigorous quality control criteria [33].

- Robustness: The ability to consistently produce reliable results with minimal variability.
- Reproducibility: The ability to get similar results over time and with different batches provided. This includes ensuring the assay’s performance is stable over time and that it can consistently detect positive and negative control chemicals.
- Rigorous procedures for procuring, handling, and verifying the purity of the chemicals being tested ensuring that any observed effects are due to the intended chemical.
- Relevance: A clear link between the biological target of the assay and a known mechanism of toxicity.
- Data interpretation using the comparison with the Toxicity Reference Database (ToxRefDB), a comprehensive database of animal toxicity studies which allowed the direct comparison of the in vitro bioactivity profile with known health effects in animals.

Additional considerations for nanomaterials testing should be considered for in vitro testing, for example,

- Characterisation of the test material in the relevant medium. Assessment of agglomeration, sedimentation or dissolution changes exposure during the assay.
- Delivery of the actual dose: The nominal concentration added to a well may not reflect the amount reaching cells.
- Assay interference: Inclusion of appropriate cell-free and material-only controls, as well as controls for interactions with dyes, probes, reagents and optical readouts.

#### 2.1.2. Mechanistic Understanding for Chemical and Material Redesign

As innovation progresses and more information becomes available, more sophisticated NAMs can support mechanistic understanding and guide molecular or process redesign through two complementary aspects (Figure 1). First, advanced NAMs can elucidate the biological pathways and mechanisms affected by new materials by identifying how specific material properties—such as size, shape, surface chemistry, solubility, persistence, or composition—influence biological responses including oxidative stress, inflammation, genotoxicity, endocrine disruption, immune activation, and cellular uptake etc. High-content imaging, transcriptomics, metabolomics, proteomics, and other omics approaches can reveal the early molecular and cellular pathways perturbed by exposure [34].

Second, mechanistic NAMs can then be used to evaluate whether redesigned materials or modified production processes reduce or avoid activation of these concerning pathways. Such targeted testing pinpoints how a substance interacts with specific genes, enzymes, or receptors. For example, mechanistic assays such as androgen or oestrogen receptor transactivation assays can identify whether a substance activates or blocks hormone receptors, providing information that can directly inform molecular redesign. This approach has been demonstrated for endocrine-modulating chemicals such as phthalates and bisphenols, where in vitro evidence on receptor activity has helped identify structural features associated with endocrine-disrupting effects and guide the development and assessment of alternative substances (Box 2). In this way, NAMs not only improve mechanistic understanding of hazard, but also support iterative material redesign, comparison of candidate materials, and identification of safer alternatives before large-scale production and environmental release. For innovations where human exposure is highly relevant, e.g., in food or cosmetics, specific NAMs can play an important role in assessing how materials interact with biological interfaces and in characterising potential exposure pathways. Advanced in vitro models, including intestinal, lung, skin, and placental barrier systems, can be used to evaluate particle uptake, transport, accumulation, translocation, persistence, and barrier disruption [35]. Such approaches are particularly relevant where exposure may occur through inhalation, ingestion, or dermal contact, and may also provide insight into maternal transfer. Complex biological models based on human cells can further enhance the innovation process by mimicking functions of human organs and tissues, allowing for human-relevant insights into toxicological and pharmacological processes. In the pharmaceutical sector, these models are particularly important because they can overcome some of the limitations associated with species differences, providing more human-relevant data and improving the prediction of efficacy and safety early in development. This may help reduce late-stage failures in drug development [36,37]. Besides helping the optimisation of lead compounds, NAMs can also support the identification of potential off-target effects as well as subpopulations that may be more susceptible to a product’s adverse effects [38]. A mechanistic understanding is the foundation for targeted molecular (re)design, allowing for a more proactive and predictive approach to chemical innovation.

Box 2 **Mechanistic NAMs in Chemical Redesign.**

Several well-known examples demonstrate how mechanistic assays are used in redesign of compounds derive from endocrine modulating chemicals such as phthalates or bisphenols. Phthalates are a class of chemicals used to make plastics more flexible but some of them such as DEHP (di-2-ethylhexyl phthalate) are known to have anti-androgenic activity [39]. This understanding comes from in vitro assays, like the Androgen Receptor (AR) transactivation assay, which measures a chemical’s ability to activate or block the androgen receptor in a cell. This assay revealed that DEHP and its metabolites could bind to the AR and prevent it from signaling properly. Using the data from these in vitro assays, chemists designed replacement chemicals that function as plasticizer but without anti-androgenic properties. They found that certain modifications to the molecular structure of the phthalate-like compounds, such as changing the central ring or the side chains, eliminated their ability to bind to the AR. This process led to the development and widespread adoption of safer plasticizers, such as DINCH (1,2-Cyclohexane dicarboxylic acid diisononyl ester). In vitro assays confirmed that DINCH and its metabolites have a very low affinity for the androgen receptor, effectively eliminating the endocrine-disrupting hazard associated with traditional phthalates while maintaining the required performance as a plasticizer [40].

Other in vitro assays are used to redesign Bisphenol A (BPA)—a chemical used to make polycarbonate plastics and epoxy resins, found in many products like food and beverage containers and the lining of metal cans. Concerns with BPA arose because its chemical structure is similar to that of 17β-estradiol, a natural hormone in the body. In vitro assays, specifically ERα BG1Luc Oestrogen Receptor Transactivation Assay, confirmed that BPA can mimic estrogen [41]. These assays are cell-based tests that measure a chemical’s ability to bind to and activate the estrogen receptor, which then turns on specific genes. The in vitro tests demonstrated that substitutions such as BPS or BPF still exhibit endocrine modulating activities and led to the development of alternatives from entirely different chemical families, aiming to completely avoid the bisphenol structure and its associated hazards. This mechanistic data provided critical evidence that BPA specifically interfered with a key biological pathway. This understanding was the foundation for regulatory actions and the push for "BPA-free" alternatives [42]. The existence of a wide range of alternatives to BPA highlights both the success of chemical redesign and the challenges of ensuring that new chemicals are truly safer [43].

#### 2.1.3. Integration of Multiple NAMs Within an Innovation Cycle

During the innovation process, integration of multiple NAMs might become necessary when a material requires the assessment of different potential hazards [44]. Often complex materials exhibit multiple “hazard hotspots” that may arise from different parts of the material structure, degradation products, or exposure conditions. At the same time, these hazards must be balanced against the intended functionality and performance of the material. For example, a material may show excellent technical performance, while also presenting concerns related to, e.g., local irritation, genotoxicity or persistence. In such cases, separate NAMs are needed to evaluate the different mechanisms and endpoints involved. Integrating these NAMs allows developers to understand trade-offs between functionality, safety, and sustainability during the redesign phase. Integration is also needed when the application context introduces multiple potential exposure routes. Materials intended for food contact, biomedical, cosmetic, or consumer applications may require assessment of, e.g., passage across biological barriers, local or systemic effects and downstream hazard mechanisms. No single NAM can capture all these aspects reliably. Therefore, different mechanistic NAMs must be combined to provide a more complete understanding of what hazards may occur, under which exposure conditions, and whether the material can still meet its intended functional requirements. In iterative innovation workflows, integrating NAMs also support prioritization decisions by helping determine whether a material should advance towards regulatory safety testing, be redesigned to reduce a specific hazard hotspot, or be discarded due to an unfavourable balance between performance, exposure, and safety. Ultimately, NAM integration is required whenever decisions depend on combining evidence across multiple mechanisms, exposure pathways, and functional requirements within the same innovation cycle [45,46]. Figure 2 illustrates an example of a food innovation pipeline in which different NAMs address complementary mechanisms, endpoints (e.g., inflammation, genotoxicity, and systemic translocation), and exposure conditions across the different physiological environments encountered during oral and gastrointestinal passage. The resulting evidence is integrated to support prioritization, redesign, and progression towards regulatory safety testing.

Complex NAMs such as 3D tissue models, organoids, and organ-on-chip systems are becoming increasingly important in order to improve translational relevance and better mimic human physiology. These systems reproduce tissue architecture, fluid flow, cell–cell interactions, and chronic exposure conditions more realistically than conventional cell cultures. Examples include gut-on-chip systems for intestinal exposure, lung-on-chip models for airborne particles, liver microphysiological systems for metabolism and accumulation studies, and multi-organ chips simulating interactions between organs. Such advanced NAMs are particularly valuable for investigating intricate hazardous mechanisms, including chronic low-dose exposure and systemic effects, that are difficult to capture using simpler assays.

However, their application in routine innovation workflows may be constrained by challenges related to standardisation, reproducibility, throughput and resource requirements. Their use should therefore be justified by the specific information needs of the decision, considering whether the additional biological complexity provides sufficient added value compared with simpler approaches.

**Figure 2:**
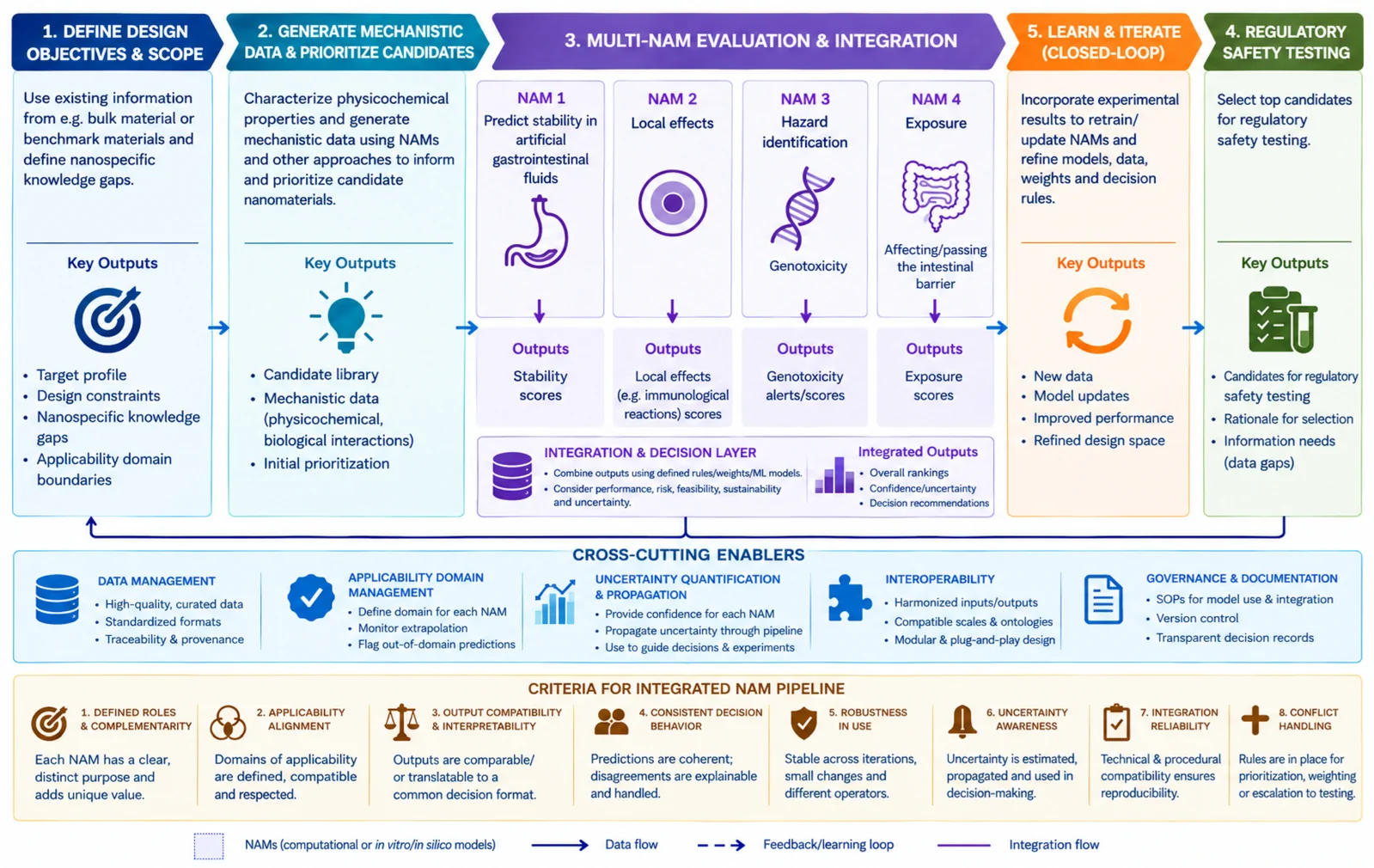
Integration of Multiple New Approach Methodologies (NAMs) into a Food Innovation Pipeline for nanomaterials.

If NAMs are integrated to provide different sources of evidence to strengthen decision-making, each NAM is not only reliable on its own, but also compatible, consistent, and decision-aligned within the shared innovation workflow where outputs can be combined without introducing instability or ambiguity.

#### 2.1.4. NAMs in Manufacturing

During manufacturing and process development, NAMs can potentially support process redesign by rapidly evaluating the biological consequences of changes in process parameters. Also, within an SSbD approach, the objective in a specific iteration cycle could be a process redesign, based on the scoping analysis of that iteration and the resulting scenario pointing towards modifying the process parameters in the context of the intended innovation [47]. Modifications to reaction conditions, raw materials, purification steps, or recycling processes may alter the formation of impurities, degradation products, residual monomers, or other non-intentionally added substances (NIAS). Combined with analytical characterisation, NAMs fulfilling context-specific requirements can provide early biological screening of these substances and help establish links between process conditions, material attributes, and biological responses (Table 1). This can support comparison of process conditions and batches, identification of safety-relevant critical quality attributes, and assessment of whether changes in processing or scale-up affect the biological activity and safety profile of the resulting material [48,49,50].

#### 2.1.5. NAMs for Assessing Environmental Hazards

Environmental safety should be considered alongside human health throughout the development of advanced materials, rather than being addressed only at the final stages of development. Environmental NAMs can provide early information on environmental fate, exposure and biological effects, complementing information generated through human-health NAMs. However, the assessment of environmental fate, exposure and ecotoxicity presents distinct methodological challenges, and the availability, applicability and maturity of environmental NAMs may vary depending on the material, environmental compartment and assessment endpoint. Depending on the stage of development and the information available, the scope and depth of environmental assessment can be progressively refined. This enables potential environmental hazards to be identified early enough to inform material selection, design and optimisation, while more detailed assessment can be undertaken as the intended use, exposure scenarios and environmental behaviour become better defined [47].

**Table 1:** Context-Specific Requirements for NAMs Across Advanced Material Innovation *.

Application	Context-of-Use	Key Objectives	Methodologies	Key Requirements
screening and prioritisation	Hazard screening Mechanistic screening Material prioritisation Comparative screening Process screening Screening for impurities and process contaminants Screening for off-target effects	Flag potential hazards early Confirm or characterise the efficacy or a known hazard mechanism Rank materials according to their biological activity, hazard potential, or suitability for further development. Compare alternative materials, formulations or designs Identify biological activity associated with unintended substances, including impurities, residuals, degradation products or contaminants. Identify unintended biological interactions or effects outside the intended function or known mechanism.	Target-based HTS (Receptor, enzyme, ion-channel, transporter assays) Reporter/pathway HTS (Receptor, stress-response and toxicity-pathway reporters) Cell-based HTS (Viability, cytotoxicity, oxidative stress, inflammation, functional assays) Phenotypic/HCS (High-content imaging, morphological profiling, cell painting) Computational screening (QSAR, read-across, molecular modelling) Analytical + biological screening (Chemical characterisation coupled to high-throughput bioassays)	High throughput Miniaturisation & low sample consumption Automation compatibility Robustness & reproducibility Sensitivity & quantitative response Biological relevance to the CoU Material/assay interference control
Mechanistic Understanding	Mechanistic elucidation Targeted molecular redesign	Identify the biological mechanisms underlying observed effects to inform targeted material redesign. Use knowledge of an identified molecular mechanism to modify the material’s molecular structure and reduce an unwanted biological effect.	Omics (transcriptomics; proteomics/phosphoproteomics; metabolomics) Molecular profiling (Cell Painting/morphological profiling; epigenetic profiling; pathway/network analysis; and targeted mechanistic assays).	AOP/mechanistic knowledge; applicability domain; biological relevance; causal interpretability; reproducibility; appropriate controls; Cost-effective and time-efficient
Supporting safety profiling	Investigate potential target organ toxicities and toxicokinetics Informing in vivo studies, Support preparation for regulatory submissions Molecular/process redesign	Identify and characterise potential target-organ toxicities and toxicokinetic profiles Inform the design, endpoints, and dosing of in vivo studies to support reduction and refinement of animal use Characterise dose–response relationships and relevant points of departure Integrate complementary NAM data to characterise the safety profile Generate and consolidate safety evidence to support regulatory submissions	Advanced in vitro models, organoids, organ-on-chip, ADME assays, PBPK/PBTK modelling Concentration–response assays, repeated-exposure models, quantitative modelling NAM batteries, integrated approaches to testing and assessment (IATA), data integration and modelling weight-of-evidence approaches, qualified NAMs and defined approaches	Reliability and assay compatibility, biological relevance, defined applicability domain, quantitative and predictive capacity, integration of complementary evidence
Optimisation of manufacturing process	Comparing manufacturing conditions Supporting process optimisation and scale-up Monitoring and controlling safety-relevant material attributes	Identify safety-relevant critical quality attributes Assess the impact of manufacturing changes on safety Establish relationships between material attributes and biological responses	Simple, targeted biological assays	sensitive to process-related changes, baseline/reference material for comparability between batches/process conditions reproducibility and material compatibility, biological relevance

* For advanced (nano)materials, physicochemical characterisation is an essential component of fit-for-purpose assessment. The parameters to be characterised should be selected according to the specific material, its intended application and exposure conditions, and the in vitro test systems used. Where relevant, this may include agglomeration and dispersion stability, protein corona formation, dissolution, biodistribution, appropriate dose metrics, and potential optical or dye-related interference with assay readouts. Characterisation should therefore be tailored to the material and the test method, rather than based on a universal set of parameters. for compliance with regulatory requirements relevant guidance should applied.

### 2.2. Establishing Confidence in NAMs for Innovation

Historically, validation has defined the process by which the reliability and relevance of a method are established for a specific purpose [51]. When used for regulatory purposes, in vitro methods were traditionally expected to undergo comprehensive validation in accordance with the principles outlined in OECD GD 34, Guidance Document on the Validation and International Acceptance of New or Updated Test Methods for Hazard Assessment [21]. This “one-size-fits-all” approach proved impractical for many NAMs used in different contexts, such as in the context of supporting decisions in innovation pipelines. Nevertheless, the foundational principles—reliability, relevance, and purpose—are applicable for NAMs used in innovation but the requirements for demonstrating these principles might differ.

- Reliability reflects the consistency and dependability of a method under its intended conditions of use. It is supported by robustness—the ability of the method to maintain performance despite small variations in operating conditions—and reproducibility, whereby comparable results can be obtained across operators, laboratories or other relevant sources of variation.
- Relevance refers to the method’s capacity to accurately measure or predict the specific effect it is intended to assess, requiring a clear, scientifically justified link to the biological endpoint of interest.
- Purpose and Context of Use (CoU) describe the overall objective of the method, while the Context of Use (CoU) defines the specific application, conditions, and limitations under which the method is considered suitable.

Establishing confidence that a new method or technology is “fit for purpose” within a company’s internal innovation pipeline requires a structured internal quality control process. Such a process provides the necessary evidence and assurance to key stakeholders, including scientists, management, and regulatory affairs teams, enabling confident decision-making throughout the innovation lifecycle (Table 2). The assessment of whether a NAM is fit for purpose should be distinguished from the decision to accept its results for regulatory purposes. During internal innovation, the developer’s scientific team is responsible for defining the context of use, establishing proportionate performance and quality criteria, documenting the applicability domain and limitations, and determining whether the available evidence is sufficient to support the intended internal decision.

Where NAM-derived evidence is intended to support a formal regulatory submission, internal assessment alone is insufficient to establish regulatory acceptability. The applicant would need to justify the scientific validity and relevance of the evidence for the regulatory question and demonstrate compliance with the applicable sector-specific requirements. The competent authority determines the acceptability of the evidence within its regulatory remit. Where an established qualification scheme is available, its procedures and evaluation criteria should be followed.

Accordingly, fit for purpose should not be interpreted as an intrinsic or universal status of a NAM. It is a judgement concerning the suitability of a method, or an integrated approach, for a specified context of use. The evidence supporting that judgement may evolve as the method, the material and the intended decision develop. This distinction allows early-stage NAMs to support innovation without implying that internal readiness constitutes formal regulatory qualification or acceptance.

By validating in-house, companies can identify and mitigate technical flaws, boundaries of applicability, or unexpected sources of variability. In particular, in vitro artefacts arising from interactions between material properties and assay components may affect the reliability and interpretation of NAM-derived data [26].

This process allows the company to compile a robust internal database of results, deepening the understanding of the method’s utility for specific tasks [52]. Crucially, the level of validation rigor is tailored to the intended use; a method deployed for rapid, high-throughput screening demands a different validation strategy than one used for final product quality testing. Ultimately, leveraging these scientific validation concepts ensures that new approach methodologies (NAMs) integrate seamlessly and reliably into existing workflows [53].

**Table 2:** Examples of existing NAM frameworks/guidance to stages of advanced material innovation and regulatory decision making.

Framework or Guidance	Main Contribution	Relevance to the Staged Approach	Important Boundary
**OECD GD 34**	Principles for validation and international acceptance of new or updated test methods.	Provides background for evaluating method reliability and relevance when used for regulatory purposes	Not a universal checklist that every exploratory NAM must satisfy.
**OECD GD 211**	Standardised description of non-guideline in vitro methods and information needed to interpret their data	Particularly relevant to early screening, mechanistic studies and documenting applicability limits.	Describing a method adequately does not itself qualify it for regulatory use.
**OECD GIVIMP**	Good scientific, technical and quality practices for in vitro methods, including SOPs, controls, performance, reporting and record keeping	Supports reliable method development and implementation across innovation stages.	Good practices support data quality but do not, by themselves, establish regulatory acceptance.
**ICCVAM** **guidance and** **reports**	Evaluation and implementation of alternative methods within the US regulatory context; relevant reports discuss evidence needs and use of NAMs.	Helps contextualise method evaluation and the use of fit-for-purpose evidence for particular decisions.	Its recommendations should not be treated as automatic acceptance criteria for other jurisdictions or sectors.
**EFSA NAMS4NANO qualification framework**	Expert-driven evaluation of scientific validity for a defined context of use, particularly for nanomaterial safety in the food and feed sector	Particularly relevant to later-stage assessment, where non-guideline NAMs may be considered for regulatory applications.	Sector-specific and distinct from OECD Test Guideline validation. The 2024 report proposed a system; it should not be represented as universal regulatory approval.
**IATA and weight of** **evidence** **approaches**	Integrate complementary information from multiple sources to address a defined assessment question.	Relevant when a single NAM is insufficient and evidence must be combined across mechanisms, endpoints or exposure pathways.	An integrated approach needs justified decision rules and an assessment of uncertainty; combining several methods does not automatically confer qualification.
**Regulatory-** **sector** **requirements**	Determine what evidence and method status are acceptable for a specific submission or decision.	Governs the transition from internal use to formal regulatory use.	Acceptance depends on the applicable legislation, sector, intended claim and competent authority.

Beyond scientific considerations, the implementation of NAMs in innovation workflows also depends on access to appropriate expertise, resources and testing infrastructures. Barriers related to infrastructure accessibility, costs and technical capacities, particularly for SMEs, have been recognised in recent European Commission policy initiatives addressing advanced materials and research and technology infrastructures [54,55]. Addressing these barriers requires more than increasing the availability of specialised laboratory equipment. It calls for an interconnected set of scientific, technological and organisational capabilities that can support NAM development, qualification and practical implementation throughout the innovation process.

Several complementary aspects need to be considered when developing or strengthening such infrastructures.

Access to appropriate experimental and computational platforms is essential, including advanced in vitro systems, organoids, organ-on-chip technologies, high-content imaging, automation, modelling tools and computational resources. For nanomaterials and other advanced materials, particular attention should be given to the integration of physicochemical characterisation with exposure-related measurements and biological testing. The selection, applicability and interpretation of NAMs depend on adequate knowledge of the materials being investigated, including their physicochemical characteristics and behaviour under relevant experimental conditions. Parameters such as particle size and size distribution, surface properties, agglomeration, dissolution and interactions with biological media may influence exposure and biological responses. Consequently, access to biological testing platforms alone may be insufficient. Integrated infrastructures that connect material characterisation with biological testing will support more reliable interpretation of results and facilitate the selection and qualification of NAMs for different materials and contexts of use. Such capabilities are particularly relevant for advanced materials, whose properties, transformations and applications may vary considerably across industrial sectors.

Equally important is the availability of reliable data infrastructures supporting the generation, management, integration and reuse of NAM-generated evidence. These include well-characterised reference datasets, common data and metadata standards, interoperable platforms and appropriate mechanisms for data access and protection. Such capabilities are particularly important when combining evidence from different experimental and computational methodologies, including AI-based approaches. Data interoperability and appropriate data management can facilitate the comparison of results, the development of predictive models and the reuse of existing evidence, while respecting applicable confidentiality and intellectual property requirements.

Another essential dimension is the availability of dedicated capabilities for method characterisation, performance evaluation and fit-for-purpose qualification. These may include access to reference/representative materials and benchmark datasets, standard operating procedures, quality assurance systems and, where appropriate, interlaboratory studies to evaluate reproducibility and transferability. Importantly, the extent of such evaluation should remain proportionate to the intended context of use and the decisions supported by the NAM, rather than imposing the same qualification requirements on all methodologies. In this respect, infrastructures should be capable of supporting different levels of method evaluation, from early-stage innovation and internal product development to applications requiring more extensive evidence for regulatory decision-making. By bringing together specialised expertise, harmonised protocols, reference materials and coordinated performance evaluation, such infrastructures can also help bridge the gap between fit-for-purpose qualification and formal standardisation, where appropriate, facilitating the transition of NAMs initially developed for innovation towards broader industrial application and regulatory acceptance.

The transition from proof-of-concept methodologies to their routine application in industrial innovation workflows also deserves particular attention. NAMs that perform well in research settings may still face challenges related to standardisation, reproducibility, throughput, automation, operational costs and scalability. These considerations are particularly relevant for complex biological systems, such as organoids and organ-on-chip technologies, whose scientific sophistication may be accompanied by demanding technical and operational requirements. Shared technology platforms, demonstration facilities and testbeds can support the evaluation of these aspects under conditions relevant to their intended application. Such facilities can also help determine whether the additional complexity of a methodology is justified by its performance and the information needed for a particular decision.

Beyond technological capabilities, the effective functioning of such infrastructures depends on accessibility, specialised skills and long-term sustainability. Establishing advanced facilities alone does not ensure that innovators, particularly SMEs, can effectively use them. Access to multidisciplinary expertise, training, technical support and sustainable service models is equally important. Greater coordination between existing research infrastructures, technology centres, industry and regulatory authorities could help reduce fragmentation, facilitate knowledge exchange and support the identification of relevant evidence needs early in material development. Rather than necessarily establishing new facilities, strengthening connections between existing infrastructures and making their complementary capabilities accessible to innovators may offer opportunities to support NAM uptake.

Existing European initiatives already illustrate different approaches to providing access to such complementary capabilities. For example, the JRC Nanobiotechnology Laboratory provides material-driven, cross-sectoral capabilities for physicochemical characterisation of nanomaterials and advanced materials and the investigation of their interactions with biological systems. This combination allows to speed up the development\qualification of methods relevant for both material design and regulatory decision. At the same time this infrastructure is accessible through its open access programme both for researchers and companies to test their materials [56]. Its participation in CERIC-ERIC [57], a distributed European research infrastructure offering coordinated access to complementary analytical facilities, further illustrates how specialised capabilities can be connected across institutions. In parallel, EU-funded Open Innovation Test Beds [58] have developed approaches to facilitating industrial access to complementary capabilities for advanced materials development, characterisation, testing and upscaling. Although these initiatives differ in scope, organisation and access conditions, they illustrate how existing research and technology infrastructures can support different stages of materials innovation and method development and provide specialised resources that small companies may not be able to maintain internally.

Regulatory sandboxes, increasingly promoted within EU innovation policy, may provide an additional mechanism for supporting NAM-based innovation by combining experimentation under regulatory supervision with opportunities for interaction between innovators and competent authorities. Depending on their design, they may also facilitate access to testing facilities and research and technology infrastructures, thereby connecting scientific and technical capabilities with regulatory learning and the identification of relevant evidence requirements [16,17,18,19,20].

Taken together, these considerations suggest that the effective use of NAMs requires not only fit-for-purpose scientific evaluation, but also accessible and interconnected infrastructures capable of supporting material characterisation, method development, qualification and application across different innovation contexts. Such infrastructures should therefore be considered an integral part of enabling NAM-based innovation, rather than solely as facilities for testing or regulatory validation.

#### 2.2.1. Readiness for Early-Stage Screening

For an in-house screening assay, besides relevance the readiness criteria focus primarily on reproducibility and the assay’s ability to reliably detect the intended biological response. The objective is to quickly and affordably identify and discard poor candidates before investing significant resources into more complex, expensive research, incl. animal studies. Biological relevance is typically demonstrated using a small training set of known positive and negative benchmark compounds. Because the specific context-of-use is early-stage screening, a flexible, fit-for-purpose validation approach is accepted. The ICCVAM report [53] specifies that lower information demands are acceptable for such screening assays, provided they maintain:

- Sufficient sensitivity to reliably detect potential hazards;
- Acceptable reproducibility to ensure confident initial prioritization;
- Clear performance metrics so that internal uncertainty is well understood;
- Transparent reporting of assumptions, data sources, and model limitations.

#### 2.2.2. Readiness for Mechanism Informed Redesign

A screening assay acts as a funnel to rapidly filter out unsuitable candidates based on simple “yes/no” or ranking metrics. In contrast, a mechanistic redesign NAM is a precision tool used to guide fine-tuned structural optimizations, requiring the model to accurately predict how small molecular changes in material structure, composition, or other properties may affect performance, stability or safety. In the future we will need more of such kinds of mechanistic assays that can support the redesign of advanced materials. Their readiness or applications in the innovation pipeline is primarily assessed by how effectively the model supports internal decision-making and iterative design cycles. The central requirement is its ability to consistently and efficiently improve molecular design outcomes within a defined material space. Other scientific/technical criteria address:

- Predictive performance (decision-relevant accuracy)

The NAM should reliably support the ranking and discrimination of redesign candidates with respect to the properties of interest, such as performance, stability, or safety. While perfect absolute prediction accuracy is not required, the model must consistently distinguish better from less suitable candidates in a way that improves design decisions, for example by increasing experimental success rates or accelerating convergence toward target properties.

- Scope and applicability domain

The NAM must operate reliably within a clearly defined material space, corresponding to its intended redesign domain. Its boundaries of applicability should be understood and respected. Inside this space, predictions should be reliable, while outside it the model may be unreliable or should not be used.

- Reproducibility and robustness

The NAM should produce stable and reproducible outputs under repeated use and within iterative design workflows. Predictions should not show uncontrolled drift over time or be unduly sensitive to minor variations in procedural conditions. Importantly, meaningful changes in material structure or composition should result in consistent and physically plausible changes in predicted properties, supporting reliable iterative optimisation.

#### 2.2.3. Readiness of a Multi-NAM System

When multiple NAMs are integrated into a single workflow, the compatibility, complementarity, and performance of the individual NAMs within the integrated system should be demonstrated [45]. The integration of results from different NAMs requires consideration of whether the methods provide complementary information or address comparable biological effects. Apparent discrepancies may arise from differences in biological endpoints, applicability domains, exposure conditions, dose metrics, or methodological limitations. Such differences should be considered when interpreting the overall evidence, rather than assuming that all NAM outputs can be directly compared or assigned equal weight [59]. The relevance and reliability of individual results, together with their associated uncertainties, influence confidence in the integrated assessment. Where important uncertainties or inconsistencies remain, these may indicate the need for additional evidence or refinement of the material or testing approach, while sufficiently robust and relevant evidence may support progression to subsequent stages of innovation. The level of confidence required depends on the intended use of the integrated evidence and the consequences of the decision it is intended to support. The integration of NAM-derived evidence is also receiving increasing attention in regulatory science. Ongoing activities at the US Food and Drug Administration (FDA) [60] and the European Food Safety Authority (EFSA) aim to facilitate the use of NAMs in regulatory assessments, including consideration of their scientific reliability, relevance, context of use and integration with other sources of evidence. EFSA is further exploring approaches for integrating NAM-derived information within next-generation risk assessment, including weight-of-evidence approaches and integrated approaches to testing and assessment (IATA) [61,62]. More specifically, the EFSA-funded NAMs4NANO project has developed a proposed context-of-use-based qualification system for NAMs and is investigating their application and integration in nanomaterial risk assessment through case studies. These activities illustrate the ongoing transition towards more flexible and scientifically justified integration of NAM-derived evidence, while highlighting the need for further experience and harmonisation to support broader regulatory application [63].

- Compatible applicability domains

The applicability domains of individual NAMs should be clearly defined and sufficiently aligned to ensure that their combined use is scientifically justified. Gaps or mismatches between applicability domains should be identified and considered.

- Defined functions and complementarity

Each NAM should have a clearly defined role within the integrated system, with its contribution to the overall assessment explicitly specified. Overlapping or redundant functions should be avoided unless they are intentionally used to strengthen confidence through independent lines of evidence.

- Transparent decision logic

The approach for combining NAM outputs should be predefined, transparent, and scientifically justified. Depending on the intended use, this may involve sequential, weighted, gated, or consensus-based integration. The decision rules should clearly describe how individual results contribute to the overall conclusion.

- Reproducibility and system stability

The integrated system should produce consistent and reproducible outcomes when applied under defined conditions and across repeated assessment cycles. Changes in individual NAM performance or decision rules should be monitored to ensure that they do not lead to uncontrolled changes in the overall outcome.

- Uncertainty and error propagation

Sources of uncertainty associated with individual NAMs and their integration should be characterised. The potential for uncertainty or error to propagate through the workflow should be assessed and considered when interpreting the overall outcome.

- Harmonised procedures and data integration

Experimental procedures, particularly sample preparation and handling, should be sufficiently aligned to ensure that results from different NAMs are comparable and can be meaningfully integrated. Data formats, processing steps, and metadata should support traceability and consistent interpretation.

#### 2.2.4. Regulatory Submission Readiness

The considerations proposed in this perspective primarily address the use of NAMs for decision-making during innovation, particularly at earlier stages of development, where NAMs can provide information to guide the development of safer and more sustainable solutions. At this stage, NAMs that are not covered by established regulatory test guidelines, such as OECD Test Guidelines, may nevertheless provide valuable information. Such NAMs are also called non-guideline NAMs. Their suitability should therefore be considered in relation to the intended decision, taking into account their scientific and technical readiness, limitations, uncertainties, and fitness for the specific purpose.

Importantly, many of the criteria relevant for the regulatory acceptance and qualification of NAMs overlap with the information needed to judge their usefulness during innovation. For example, understanding the biological relevance, reliability, reproducibility, applicability domain, limitations, uncertainties, and interpretation of NAM-derived results is important both when using a NAM to inform an early innovation decision and when subsequently considering its use in a regulatory context. The distinction therefore does not necessarily lie in the criteria themselves, but rather in the required level of evidence, certainty, standardisation, and regulatory acceptance. The same criteria can thus provide a basis for assessing NAM readiness throughout the innovation process, while the expectations associated with their application become more stringent as decisions approach a regulatory context.

As innovation progresses, NAMs may increasingly be used in contexts with direct regulatory relevance. For example, a non-guideline NAM that has demonstrated sufficient reliability and relevance during innovation may subsequently be considered for regulatory submission as supporting evidence, to support a waiver of an animal study, or to inform the design of in vivo studies. NAMs used to guide subsequent in vivo studies should likewise provide sufficiently reliable and relevant information to support the design and justification of those studies. At this stage, the evidence generated by the NAM needs to be considered against the requirements of the relevant regulatory framework.

The use of NAMs in regulatory decision-making, including their validation and qualification requirements, has been widely discussed elsewhere [64,65,66,67] and is therefore not reviewed in detail here. Recent initiatives illustrate the ongoing development of pathways for the regulatory use of non-guideline NAMs. For example, the NAMs4NANO project funded by the European Food Safety Authority has proposed a framework for the qualification of non-guideline NAMs in the food sector [23,67]. Emerging regulatory approaches, such as the regulatory sandboxes proposed under the EU Biotech Act, may further provide opportunities to explore the use of NAMs under regulatory oversight.

These developments highlight an important distinction for the framework proposed here. The readiness criteria should not be interpreted as prerequisites for the early use of NAMs during innovation, nor as an alternative to regulatory qualification. Rather, they provide a means of assessing whether a NAM is sufficiently fit for its intended use at a given stage of innovation, while making its limitations and uncertainties explicit. As development progresses towards regulatory submission, developers will need to transition towards methods and evidence that satisfy the applicable regulatory requirements. Thus, the criteria relevant to regulatory acceptance can already inform the assessment of NAMs during innovation, but the level of evidence and certainty required for regulatory decision-making will generally be higher.

## 3. Discussion

The increasing complexity of advanced materials is transforming innovation from a largely linear process into an iterative cycle of design, testing and redesign. Materials innovation is characterised by iterative design cycles in which decisions must often be made under conditions of incomplete knowledge and evolving material characteristics. In contrast, conventional safety assessment approaches have traditionally been applied at later stages of development, limiting their ability to guide early design choices. This has motivated the development of proactive approaches, such as Safe-and-Sustainable-by-Design, which integrate safety (and also sustainability) considerations earlier in the innovation process to support informed decision-making while sufficient design flexibility remains [54,55,68,69].

Fit-for-purpose NAMs, with the level of evidence and readiness tailored to the intended use and applicability domain of the NAM, could efficiently accelerate the innovation process for advanced materials. By providing earlier, mechanistic, and human-relevant information, NAMs can support decision-making throughout the innovation pathway. Their value lies not only in their potential to replace traditional in vivo approaches, but also in their ability to generate decision-relevant evidence at critical decision points where choices regarding material selection, optimisation, and further development are made. Importantly, however, the information required to support these decisions is not the same at every stage of innovation. Different decisions involve different consequences and levels of uncertainty and therefore require different degrees of confidence in the evidence.

This context-dependent approach shifts the focus from whether a NAM is universally “validated” towards whether the available evidence is sufficient for the specific decision needed and context of use. Confidence in a NAM can instead be built progressively as evidence accumulates for a defined application. This concept of flexible, purpose-driven qualification is supported by scientific-technical criteria described in OECD Guidance Document 211 for the use of non-guideline methods as well as approaches proposed for, e.g., developmental neurotoxicity [70,71,72] and the for the food sector [22]. Early applications may rely on evidence demonstrating technical feasibility, mechanistic understanding and biological relevance, whereas applications requiring higher confidence demand stronger evidence related to reproducibility, predictive performance, robustness and transferability. Such a fit-for-purpose approach ensures that evidence generation is proportionate to the intended use, prioritises the evaluation of uncertainties relevant to the decision at hand, and supports efficient use of resources by avoiding excessive or unnecessary validation exercises. These different evidence requirements are reflected across the innovation lifecycle. At early stages of development, screening-level NAMs can support rapid prioritisation of candidate materials by identifying desirable and undesirable properties before substantial resources are invested. Greater uncertainty may be acceptable at this stage because decisions remain relatively reversible and can be refined through subsequent testing. As development progresses, more advanced in vitro and omics-based approaches can provide mechanistic insights that support the redesign of materials to eliminate hazardous mechanisms while preserving functionality. Further downstream, NAMs may also support manufacturing and quality-control activities, where sensitivity, robustness, and reproducibility become increasingly important for identifying impurities, transformation products, or other changes in material characteristics. Thus, the role and evidence requirements of a NAM may evolve alongside the material and the decisions it is intended to support.

## 4. Conclusions

The main difference between an in-house NAM and a regulatory accepted NAM is not necessarily the underlying science, but the context-of-use, evidence requirements, and tolerance for uncertainty. An in-house NAM is built to support internal design decisions, such as prioritisation candidate materials or comparing alternatives during early optimization. In this context, utility, speed, and the ability to provide a reliable relative ranking may be more important than highly accurate absolute predictions, particularly when decisions are reversible and experimental confirmation can follow.

In contrast, regulatory NAMs are intended to support formal safety, compliance, or risk-assessment decisions that must withstand external scientific and regulatory scrutiny. Consequently, they require a substantially higher level of confidence, supported by evidence of reproducibility, robustness, predictive performance, transferability, and clearly defined applicability domains.

The integration of NAMs into advanced-material innovation should therefore not be viewed as a choice between “validated” and “non-validated” methods. Instead, confidence should be built progressively and proportionately to the intended use and the consequences of the decision being supported. Accelerating innovation does not require less evidence; it requires the right evidence, generated at the right time and at a level of confidence appropriate to the decision. A fit-for-purpose approach to NAM qualification can therefore help integrate scientific confidence into iterative material development while ensuring that evidence requirements increase appropriately as decisions become less reversible and more consequential.

## Figures and Tables

**Figure 1:**
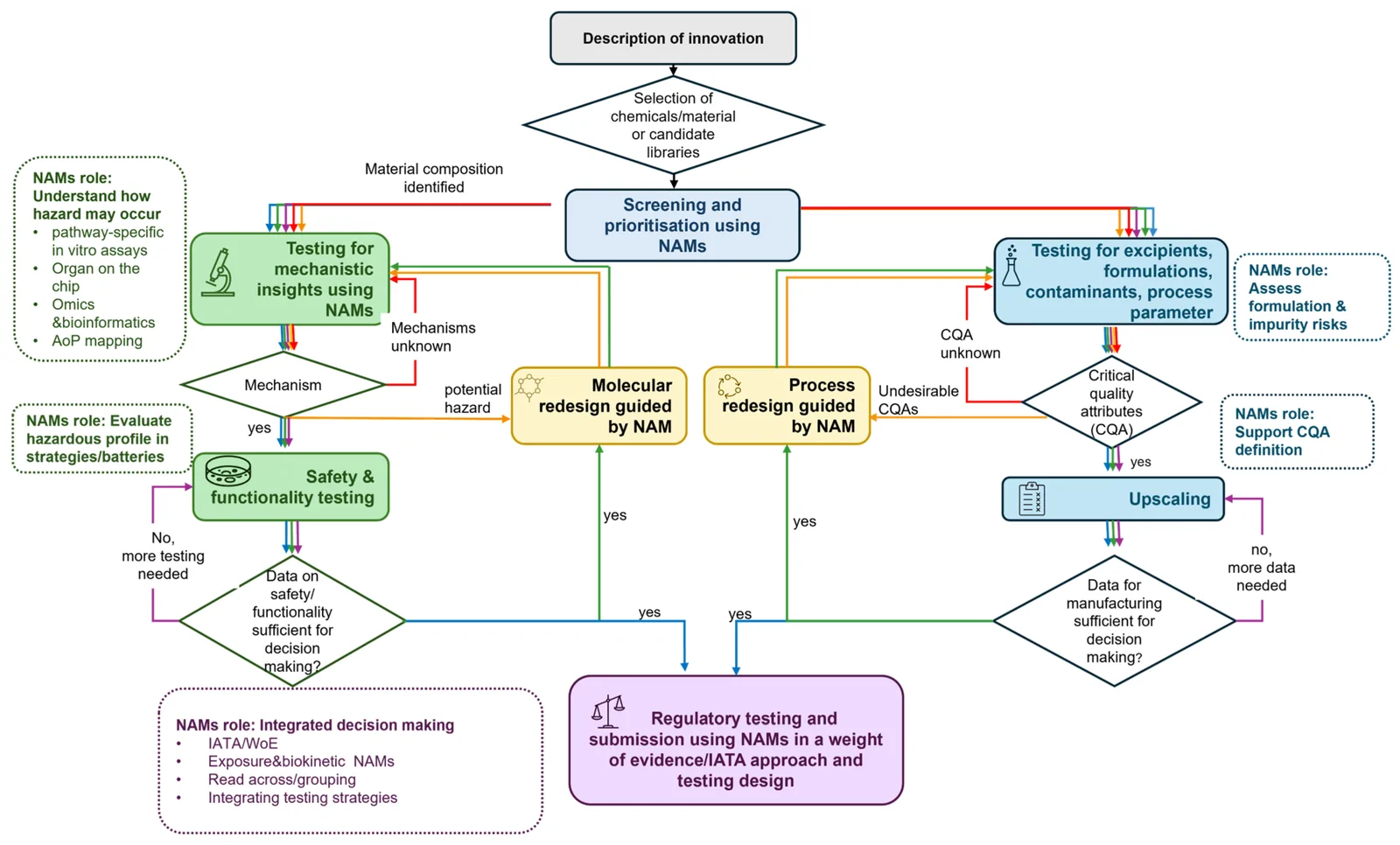
Potential contributions of NAMs in innovation.

## Data Availability

No new data were created or analyzed in this study. Data sharing is not applicable to this article.

## Abbreviations

The following abbreviations are used in this manuscript:

CoU	Context of use
EC	European Commission
GD	Guidance Document
IP	Intellectual property
NAM	New Approach Methodology
OECD	Organisation for Economic Co-operation and Development
NAMs4NANO	Integration of New Approach Methodologies results in chemical risk assessments: Case studies addressing nanoscale considerations
SSbD	Safe and Sustainable by Design
